# The Need for Sustainability, Equity, and International Exchange: Perspectives of Early Career Environmental Psychologists on the Future of Conferences

**DOI:** 10.3389/fpsyg.2022.906108

**Published:** 2022-06-16

**Authors:** Jana K. Köhler, Agnes S. Kreil, Ariane Wenger, Aurore Darmandieu, Catherine Graves, Christian A. P. Haugestad, Veronique Holzen, Ellis Keller, Sam Lloyd, Michalina Marczak, Vanja Međugorac, Claudio D. Rosa

**Affiliations:** ^1^Urban and Environmental Psychology Group, Department of Cognition, Emotion, and Methods in Psychology, Faculty of Psychology, University of Vienna, Vienna, Austria; ^2^Transdisciplinarity Lab, Department of Environmental Systems Science, Institute for Environmental Decisions, Eidgenössische Technische Hochschule Zurich, Zurich, Switzerland; ^3^ECM-LIREM, EA4580, Univ. Pau & Pays Adour/E2S-UPPA, Bayonne, France; ^4^Facultad de Economía y Empresa, Universidad de Zaragoza, Zaragoza, Spain; ^5^Sustainability Research Institute, School of Earth and Environment, University of Leeds, Leeds, United Kingdom; ^6^Department of Psychology, University of Oslo, Oslo, Norway; ^7^Department of Environmental Psychology, Institute of Psychology, Otto-von-Guericke-University Magdeburg, Magdeburg, Germany; ^8^School of Psychology and Computer Science, University of Central Lancashire, Preston, United Kingdom; ^9^Department of Psychology, University of Victoria, Victoria, BC, Canada; ^10^Institute of Psychology, Norwegian University of Science and Technology, Trondheim, Norway; ^11^School of Business, University College Dublin, Dublin, Ireland; ^12^Department of Development and Environment, Universidade Estadual de Santa Cruz, Ilhéus, Brazil

**Keywords:** scientific conferences, early career researcher (ECR), travel-intensive conferences, academic travel, equitable academia, accessibility, inclusivity, sustainable academia

## Abstract

At the 2019 and 2021 International Conference on Environmental Psychology, discussions were held on the future of conferences in light of the enormous greenhouse gas emissions and inequities associated with conference travel. In this manuscript, we provide an early career researcher (ECR) perspective on this discussion. We argue that travel-intensive conference practices damage both the environment and our credibility as a discipline, conflict with the intrinsic values and motivations of our discipline, and are inequitable. As such, they must change. This change can be achieved by moving toward virtual and hybrid conferences, which can reduce researchers’ carbon footprints and promote equity, if employed carefully and with informal exchange as a priority. By acting collectively and with the support of institutional change, we can adapt conference travel norms in our field. To investigate whether our arguments correspond to views in the wider community of ECRs within environmental psychology, we conducted a community case study. By leveraging our professional networks and directly contacting researchers in countries underrepresented in those networks, we recruited 117 ECRs in 32 countries for an online survey in February 2022. The surveyed ECRs supported a change in conference travel practices, including flying less, and perceived the number of researchers wanting to reduce their travel emissions to be growing. Thirteen percent of respondents had even considered leaving academia due to travel requirements. Concerning alternative conference formats, a mixed picture emerged. Overall, participants had slightly negative evaluations of virtual conferences, but expected them to improve within the next 5 years. However, ECRs with health issues, facing visa challenges, on low funding, living in remote areas, with caretaking obligations or facing travel restrictions due to COVID-19 expected a switch toward virtual or hybrid conferences to positively affect their groups. Participants were divided about their ability to build professional relationships in virtual settings, but believed that maintaining relationships virtually is possible. We conclude by arguing that the concerns of ECRs in environmental psychology about current and alternative conference practices must be taken seriously. We call on our community to work on collective solutions and less travel-intensive conference designs using participatory methods.

## Introduction

International scientific conferences held in person are unsustainable and inequitable. Attendee travel (among other factors) causes high CO_2_ emissions thus contributing to climate change (van Ewijk and Hoekman, 2021). Unsurprisingly, air travel makes up a substantial share of many academic institutions’ overall emissions (e.g., 63–73% at the University of British Columbia; Wynes and Donner, 2018), of which conference travel constitutes a substantial share (Achten et al., 2013). Further, the requirement for traveling to conferences systematically excludes disadvantaged researchers [e.g., researchers with disabilities, caretaking responsibilities, early career researchers (ECRs); Sarabipour et al., 2021; Skiles et al., 2021].

In this community case study, we focus on travel-intensive conference practices within the environmental psychology research community. We suggest that, in addition to researching air travel behavior, the community should reduce its own air travel. This change, however, is difficult, as academics often regard air travel as necessary to advance their career (e.g., to network, to disseminate their work; Higham et al., 2019; Nursey-Bray et al., 2019), even though evidence suggests that air travel influences academic career success only limitedly (Wynes et al., 2019).

Against this backdrop, symposia took place at the 2019 and 2021 International Conference on Environmental Psychology (ICEP), during which researchers debated how the environmental psychology community should conduct conferences in the future. Many community members agreed that emissions from conference travel should be reduced. Nevertheless, some cautioned against changing current conference practices, fearing this could undermine the quality of the conference. In this manuscript, we—an international author team of ECRs in environmental psychology—wish to continue this timely debate by contributing an ECRs perspective. We continue to focus on conference travel, as some suggest that conferences are the most common purpose for academic travel (Glover et al., 2019). Conferences also offer an opportunity to alter the behavior of many individuals at once by fostering new community norms.

## Conference Practices in the Environmental Psychology Community

Conferences on environmental psychology have served our community by fostering interconnectedness and research collaboration. These conferences have evolved over the years to adapt to changing circumstances and shifting needs of a developing research community (e.g., increased internationalization), and they must continue to adapt in order to address environmental and equity challenges. Some steps in this direction have already been taken. For example, when submitting bids to host ICEP, bidders must provide information on the carbon emissions associated with their proposal (ICEP Organizing Committee, 2019).

As ECRs, we are very grateful for the immense effort that was invested into designing and maintaining conferences like ICEP. Nevertheless, we believe that in light of the emissions and inequities associated with travel-intensive conference practices, it is time to adapt further, not least to accommodate the growing number of community members who want to reduce their travel emissions to limit global warming (Together Science Can, 2019; Matthies et al., 2021) as well as disadvantaged researchers who are currently excluded from conferences. We do not wish to disregard the benefits of current conference travel practices, but to invite the community to look for new approaches, which allow us to make necessary changes without losing the valuable structures we have established.

## Aim of This Manuscript

We aim to continue the discussion about the format of future conferences started at ICEP 2019 and 2021, and to develop it by providing an ECRs perspective. We further aim to provide empirical insights into the views of ECRs in our community regarding travel-intensive conference practices and their visions of possible changes. In doing so, we want to make it easier for those making strategic decisions in our community to consider the viewpoints of ECRs.

We deem it particularly important to present the views of ECRs because their views are currently underrepresented in conference planning (Bankston et al., 2020), despite the prevalent belief that they must travel in order to build a professional network (Higham et al., 2019; Matthies et al., 2021). This gives ECRs a special position within the discourse on academic conference travel, which we would like to comment on.

While we argue in favor of less travel-intensive conference practices, we do not disregard arguments cautioning against change, nor do we call for all international exchange to be conducted virtually. Further, we acknowledge that changing conference travel practices might come with disadvantages. Nevertheless, we deem change necessary to secure the continued credibility and legitimacy of environmental psychology, and invite our community to think creatively about how disadvantages can be minimized or transformed into benefits.

## Materials and Methods

In February 2022, we distributed an online survey among ECRs in environmental psychology by using our professional networks (e.g., mailing lists, Twitter, Slack) and by directly contacting researchers in countries underrepresented in those networks. We aimed to gather quantitative data on how other ECRs in environmental psychology think about the future of conferences in environmental psychology. The survey included questions about participants’ perceptions of current conference travel practices, their opinions regarding changing conference practices, and more specifically their views on virtual and hybrid conferences. Additionally, we used open-ended questions (Supplementary Tables 1–9) to elicit participants’ suggestions and ideas for less travel-intensive conference practices.

We applied particular caution not to lead participants in their survey responses. Firstly, we started the survey with general questions on current conference travel practices and different conference formats, mentioning flying in particular only later in the survey. Secondly, we interspersed more morally loaded questions with more neutral questions on the use and liking of different conference formats.

We obtained responses from 117 ECRs^1^ living in 32 countries. The majority of participants were Ph.D. students (54%) and worked on climate change-related topics as opposed to other topics in environmental psychology (81%). Overall, participants seemed to have some prior experience with conferences, having attended *Mdn* = 6.00 (range = 0–48) in-person conferences and *Mdn* = 3 (range = 0–15) virtual conferences throughout their academic career. In a typical year before the COVID-19 pandemic, 77% of participants flew to conferences once a year or less. For more demographic data see Table 1.

**TABLE 1:** Sample description.

	*N*	%	M, SD
**Gender**			
Female	67	57%	
Male	46	39%	
Non-binary	2	2%	
No answer	2	2%	
**Position**			
Postgraduate researchers not holding/doing a Ph.D.	12	10%	
Ph.D. students	63	54%	
Postdocs	23	20%	
Senior researchers/lecturers	7	6%	
Professors	12	10%	
**Conference experience**			
Never having attended an in-person conference	14	12%	
Never having attended a virtual conference	8	7%	
Age (23–50)			31.22, 5.06

Data were analyzed in IBM SPSS Statistics (IBM Corp, 2020), RStudio (RStudio Team, 2015), QDA Miner (LaPan, 2013), and NVivo (Welsh, 2002). The full questionnaire as well as the anonymized dataset can be found on the Open Science Framework.^2^ We present the results of the survey in the following Sections “Why International Conference Travel Practices need to Change” and “How International Conference Travel Practices Can be Changed,” alongside our own arguments for less travel-intensive conference practices. We chose this structure to facilitate cross-comparison between our views and those held by other ECRs in environmental psychology. Unless otherwise indicated, all variables were measured on a scale from 1 (strongly disagree) to 7 (strongly agree).

## Why International Conference Travel Practices need to Change

We believe that current travel-intensive conference practices need to change because they pose a direct threat to the environment. Mainly due to attendee travel, in-person conferences are associated with large amounts of CO_2_ emissions (van Ewijk and Hoekman, 2021). As technological advancements will likely not sufficiently reduce the aviation industry’s emissions (Grewe et al., 2021), additional behavior change is required to meet global emission reduction targets.

The survey participants also believed that flying to conferences harms the environment (*M* = 6.47, *SD* = 0.96). On average, participants wanted conference travel practices in environmental psychology to change (*M* = 5.62, *SD* = 1.45), thought flying to conferences should be reduced (*M* = 6.00, *SD* = 1.12), and perceived the number of researchers who want to reduce emissions from conference travel to be growing (*M* = 5.68, *SD* = 1.22). In their responses to open-ended questions, participants also expressed the belief that it was important for travel norms in our community to change.

More indirectly, travel-intensive conference practices also pose a risk to our scientific discipline. If we as environmental psychology researchers do not reduce our air travel, it may negatively affect our credibility when communicating the need for behavior change to the public (Le Quéré et al., 2015; Attari et al., 2016). Inconsistencies between our call for change and our behavior may be actively instrumentalized to promote discourses of climate delay (Lamb et al., 2020), and even those with no interest in delaying climate action may doubt that climate change requires urgent action if experts on this topic behave unsustainably. Importantly, criticism of sustainability researchers’ behavior is often presented in bad faith. Thus, aiming to satisfy all demands for ethical purity is futile (Goodwin, 2020). Nevertheless, we think that the environmental psychology community should act as role models by systematically addressing the largest sources of emissions in their professional carbon footprints.

In line with these arguments, participants on average somewhat agreed that flying to conferences harms our credibility (*M* = 5.12, *SD* = 1.71) and somewhat disagreed that such behavior was in line with being a role model for the public (*M* = 2.69, *SD* = 1.65; reverse-coded) or with leading by example for other scientific disciplines (*M* = 2.85, *SD* = 1.71, reverse-coded). They considered protecting the environment (*M* = 6.60, *SD* = 1.09) and the credibility of researchers in our field (*M* = 5.25, *SD* = 1.77) compelling reasons to reduce flying to conferences, on a scale from 1 (a very bad reason) to 7 (a very good reason). Being a role model for the public (*M* = 5.74, *SD* = 1.59) and leading by example for other scientific fields (*M* = 5.72, *SD* = 1.51; measured on the same scale) were also considered important.

Additionally, we experience tension between our closely held sustainability values and the behavior required by conference travel norms. This causes cognitive dissonance and thus discomfort (Festinger, 1957). Like us, many ECRs in environmental psychology are strongly motivated in their work by their wish to help avert a climate crisis. On average, the survey participants indicated that mitigating climate change (*M* = 6.65, *SD* = 0.63) as well as having low carbon footprints in their professional (*M* = 6.11, *SD* = 0.91) and private lives (*M* = 6.27, *SD* = 0.91) is important to them. When we asked participants why they became researchers or why they chose to work on environmental psychology topics in particular, “wanting to change the world for the better,” and not least “mitigating climate change” were among the most frequently stated motivations (Supplementary Tables 1, 2). Significantly, participants also believed that it was important to advance their careers (*M* = 6.03, *SD* = 1.09). Thus, ECRs in environmental psychology may find themselves in a situation of conflicting priorities.

Concerns about the negative consequences of travel-intensive conference practices were also reflected in ECRs’ emotions and norms. Participants thought their colleagues should not fly to conferences (*M* = 5.00, *SD* = 1.55), but did not perceive a similar expectation toward themselves from their colleagues (*M* = 3.89, *SD* = 1.55). On a scale of 1 (never), 2 (rarely), 3 (sometimes), 4 (often), and 5 (every time), they reported often feeling bad about their conference air travel emissions (*M* = 3.92, *SD* = 1.11), and sometimes worrying about being criticized for it (*M* = 2.81, *SD* = 1.33) although they had rarely been criticized yet (*M* = 2.20, *SD* = 1.30). They did not experience any do-gooder derogation (see Minson and Monin, 2012), reporting that they rarely got criticized for not flying to conferences (*M* = 1.87, *SD* = 1.31) and also rarely worried about it (*M* = 2.02, *SD* = 1.14).

We think that travel-intensive conference practices are in tension with the very values and moral aspirations underlying our research, at least for those working on climate change topics. Resolving such tensions as far as possible would make it easier for researchers strongly rooted in these moral concerns to work in academia. It may even retain some researchers who might otherwise leave academia for less travel-intensive careers. Although only 5% of the participants knew of someone who had left academia because they could not or did not want to keep up with travel requirements, 13% had themselves considered leaving academia for this reason.

Equally as important as the threat to the environment, current conference travel practices are associated with considerable inequities reflected in as well as perpetuated by inequalities of access to conferences. Academic air travel is strongly predicted by one’s geographic location and family commitments (Whitmarsh et al., 2020). Those facing visa issues or lacking funding for (long-distance) travel are systematically disadvantaged by in-person conferences (McInroy, 2018). Caretaking responsibilities and physical or mental disabilities or illnesses similarly inhibit travel (Bradley et al., 2020; Henderson, 2021). More recently, travel regulations put in place due to the COVID-19 pandemic (e.g., tests and vaccinations as entry requirements) may place additional travel burdens on some (Matthies et al., 2021).

**FIGURE 1:**
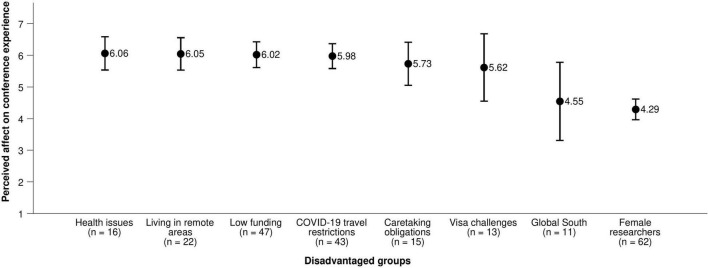
How a virtual conference format would affect the conference experience of participants’ groups. Variables were measured on a scale from 1 (it would make their experience much worse) and 7 (it would make their experience much better). Error bars represent 95% confidence intervals.

**FIGURE 2:**
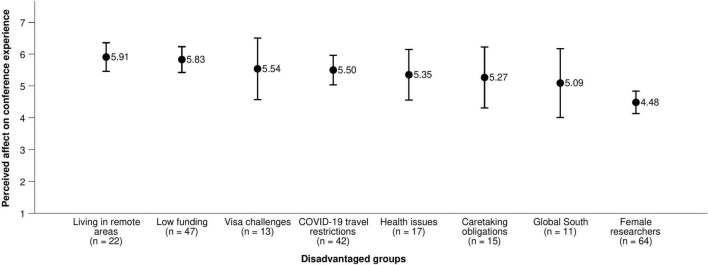
How a hybrid conference format would affect the conference experience of participants’ groups. Variables were measured on a scale from 1 (it would make their experience much worse) and 7 (it would make their experience much better). Error bars represent 95% confidence intervals.

Participants also believed that flying to conferences reinforces existing inequalities (*M* = 5.81, *SD* = 1.30). Although on average participants said they could participate in their field’s conference travel practices without major difficulties (*M* = 4.60, *SD* = 1.82), a substantial minority of 29% indicated they could not; especially researchers who indicated they were from the Global South (*M* = 2.82, *SD* = 2.32, *n* = 11^3^) or facing visa challenges (*M* = 2.85, *SD* = 1.91, *n* = 13). In contrast, participants who did not identify as members of any disadvantaged group (including all of the groups in Figures 1, 2 and any other group they wished to name) were able to participate easily (*M* = 5.80, *SD* = 1.01, *n* = 17).

## How International Conference Travel Practices Can be Changed

To us, the question is not whether conference travel practices should change, but how we should change them. Our discourse needs to shift toward best practices and creative visions of what successful, less travel-intensive conferences could look like. We do not postulate that change must happen in a specific way. Rather, we believe it will require the ingenuity of our whole community to find ways to move forward. Nevertheless, we wish to provide a few examples of less travel-intensive conference practices.

The most common alternatives to in-person conferences are virtual or hybrid conferences. While interactions take place entirely online at virtual conferences, hybrid conferences allow for both in-person and virtual participation. Hybrid conferences may be held in one central location and also allow for virtual participation, or attendees may meet in local hubs on different continents or countries and connect virtually across them (Kuper, 2019). Virtual conferences can reduce conference emissions by up to 94%, hybrid conferences by 60–70% (Tao et al., 2021). Additionally, there are other measures to minimize travel emissions from conferences (see Kreil, 2020). Train travel may be promoted by providing booking support for train trips and financial compensation (Hartmann et al., 2019). Annual international conferences could be changed to a biennial cycle or alternate between a virtual and in-person format. Conferences could be held at locations that minimize travel emissions across all attendees (Klöwer et al., 2020). Alternatives suggested by participants included: regional conferences, choosing local conference hubs that are virtually connected, incentivizing and promoting train travel, and ensuring sustainable food and accommodation (see Supplementary Table 3).

An important question is whether virtual/hybrid conferences can deliver the same benefits as in-person conferences. Answering this question is crucial because participants expressed a belief that conference travel is beneficial for their career, both in the quantitative data (*M* = 5.71, *SD* = 1.34) and in their responses to open-ended questions. Participants also expected their conference experience^4^ to become somewhat worse upon switching to virtual conference formats [*M* = 3.51, *SD* = 1.59; scale from 1 (It would make my experience much worse) to 7 (It would make my experience much better)], although they were optimistic that virtual conferences would improve within the next 5 years [*M* = 5.36, *SD* = 1.48; on a scale from 1 (not at all optimistic) to 7 (very optimistic)]. They expected a somewhat better conference experience when switching to hybrid formats (*M* = 4.53, *SD* = 1.63). When they rated the usefulness (on a scale from 1 to 10) of the best, worst, and average in-person and virtual conferences they had attended, virtual conferences were consistently rated lower than in-person ones (Table 2). However, the best virtual conferences (*M* = 6.46, *SD* = 2.15) received similar ratings as the average in-person conferences participants had attended (*M* = 6.88, *SD* = 1.39).

**TABLE 2:** Usefulness of the best, worst, and average in-person and virtual conferences participants had attended.

	In-person		Virtual	
	conferences		conferences	
	M	SD	M	SD
Best attended conferences	8.13	1.71	6.46	2.15
Average attended conferences	6.88	1.39	5.42	1.85
Worst attended conferences	4.35	2.02	3.10	1.94

*Variables were measured on a scale from 1 (extremely useless) to 10 (extremely useful).*

Commonly discussed disadvantages of virtual and hybrid conferences include time-zone differences, screen fatigue, or technical issues (e.g., Foramitti et al., 2021). However, the biggest disadvantage of virtual conferences is perceived to be the lack of informal and social exchange and chances to network (Foramitti et al., 2021; Matthies et al., 2021; Wenger, 2021). This was confirmed by our qualitative data, as formal sessions were considered easy to conduct virtually, whereas informal chats and spontaneous exchanges, socializing (e.g., during coffee or lunch breaks), networking with new people, and forming relationships were considered difficult to realize virtually (Supplementary Table 4).

However, some participants thought that informal and social interactions could indeed be facilitated virtually (Supplementary Table 5). Some said it was easy to conduct planned informal chats and small group discussions, that online tools and platforms could be used for virtual social activities such as team games, and that chat forums and other interactive platforms could help to network and foster information exchange (Supplementary Tables 5, 6). Several participants shared our opinion that difficulties around informal interaction must be carefully considered in the planning of virtual/hybrid conference formats (e.g., planned sessions for networking and informal exchange; Supplementary Table 6).

Similarly, quantitative data suggested that participants were of mixed opinions regarding their ability to build and maintain professional relationships through virtual communication, reflected in a clearly bimodal distribution of responses. On a scale from 1 (very unlikely) to 7 (very likely), 39% said it was (very or somewhat) unlikely that they could build new professional relationships virtually, while 55% said it was (very or somewhat) likely (*M* = 4.28, *SD* = 1.62). Maintaining already established professional relationships through virtual communication was considered more achievable (*M* = 5.25, *SD* = 1.39).

Prioritizing informal exchanges in virtual conference planning could help to build and maintain strong bonds with each other. Our community already has great structures and networks in place, which have been built over the past years and from which ECRs can now benefit (e.g., there are many local networks, events, and conferences, albeit with large geographical inequalities). Thanks to recent technological developments as well as conference organizers’ creativity, several formats have emerged that address perceived shortcomings of current virtual conferences (Song et al., 2021; see https://thefutureofmeetings.wordpress.com/tools/ for a list of tools for virtual communication). One particularly noteworthy recent example of such formats is a mentoring program that paired junior researchers with senior researchers based on their interests, aiming to have informal virtual chats throughout the conference to network (Sips Society for the Improvement of Psychological Science [SIPS], 2021).

### New Formats Provide an Opportunity to Reduce Existing Inequalities and Promote Equity

Switching to alternative conference formats could at least partially alleviate travel burdens experienced disproportionally by the disadvantages mentioned in the Section “Why International Conference Travel Practices Need to Change.” Virtual conferences increase attendance and diversity, especially with respect to gender, geographic location, and career stage (Sarabipour, 2020; Skiles et al., 2021). For virtual and hybrid conferences, most attendees benefit from savings in time and money (Sarabipour, 2020; Foramitti et al., 2021). Researchers with disabilities (e.g., deaf and hard of hearing), an often-overlooked disadvantaged group, can benefit from unique features of virtual conferences such as the addition of subtitles to recorded talks (Huyck et al., 2021). Virtual conferences may also increase ECRs’ active participation through reducing social anxieties (Estien et al., 2021). In line with this, participants considered reducing inequalities a compelling reason to change current conference travel practices [*M* = 5.89, *SD* = 1.44, on a scale from 1 (a very bad reason) to 7 (a very good reason)].

Nevertheless, new conference formats may also introduce or exacerbate inequalities. For example, the funding available to a researcher might dictate whether they attend a given hybrid conference virtually or in person. For some, virtual participation may be the only possibility to participate at all. Vice versa, the mere option of inexpensive virtual participation might keep institutions from funding costlier in-person participation, thus also preventing some from attending in person. This might introduce systematic differences in the quality of the conference experience for different groups of researchers. Similarly, a conference location that minimizes the total travel emissions across all attendees would systematically disadvantage those living in more remote locations. We do not condone such effects, and urge the community to focus on developing alternatives which improve, rather than impair, equality of access.

In practice, the positive and negative impacts of changing conference travel practices for specific groups in specific circumstances must be carefully evaluated and weighed. Our survey data supports this view. Participants with health issues, visa challenges, caretaking obligations, COVID-19 travel restrictions, low funding, or living in areas remote from centers of academic activity thought that the conference experience of researchers in their own disadvantaged group would improve through switching to virtual or hybrid conferences (Figures 1, 2). However, it is noteworthy that the same participants did not necessarily expect positive impacts for themselves to the same extent (Supplementary Figures 1, 2).

### Collective Solutions Can Change the Rules of the Game and Create Equal Conditions for All

We believe that many actors in academia have a role to play in this transformation toward less travel-intensive conference practices, and that the responsibility can neither be put solely on individuals nor solely on collective entities. In accordance with this view, participants considered changing conference travel practices a shared responsibility, with themselves (*M* = 5.71, *SD* = 1.18), the research community (*M* = 5.93, *SD* = 1.07), conference organizers (*M* = 6.03, *SD* = 1.02), and employing institutions (*M* = 5.48, *SD* = 1.47) responsible for making changes. In the open-ended “Other” category, participants indicated that they also considered governments and states (*n* = 8), policymakers (*n* = 7), and funding agencies (*n* = 5) responsible (Supplementary Table 7).

Here, it is crucial to emphasize the particular importance of collective and institutional changes. Both are needed to support individual behavior change by mitigating negative consequences of individual behavior change. For example, in discussions of academic air travel, many researchers express concern for the career opportunities of ECRs who choose not to fly, or would not be able to, if we collectively, as a discipline, decided not to fly.

Indeed, participants expected to be put at a slight disadvantage compared to researchers at their own career stage (*M* = 4.87, *SD* = 1.59) and further along the career path (*M* = 4.96, *SD* = 1.59) if only they themselves chose to fly less to conferences. However, in line with our argument above, participants did not think that they would be put at a disadvantage if everyone in the field flew less, both compared to those at their own career stage (*M* = 2.76, *SD* = 1.49) and those at higher career stages (*M* = 3.19, *SD* = 1.70). This underlines the importance of seeking collective solutions. We recognize the unique role of conference organizers as a relevant stakeholder group to initiate collective change in conference travel practices. However, the burden of change should not be placed exclusively on conference organizers. They should be supported in change by other relevant stakeholders like universities, professional associations of psychologists, and external stakeholders (e.g., sponsors, or municipalities where conferences are held).

## Discussion

We have presented arguments in favor of switching to less travel-intensive and thus more sustainable and equitable conference practices in environmental psychology. Additionally, we present empirical insight into what the international community of ECRs in environmental psychology thinks on this topic.

Participants believe that conference travel practices should change, and that flying to conferences should be reduced in particular. This belief may stem from their sustainability values, which motivated many to work on environmental psychology, or from their difficulties in complying with current travel norms. Participants consider reducing flying necessary to protect the environment and promote equity by reducing inequalities in academia, but also to preserve our community’s credibility and ability to lead by example. A minority of the participants has even considered leaving academia because they could not or did not want to comply with travel practices. This, and the fact that the participants perceive the number of researchers wanting to reduce their travel emissions to be growing, should give us pause for thought.

The ECRs we surveyed consider conferences beneficial for their careers. Thus, we want to emphasize that abolishing conferences or conference travel is not our intention. Instead, we advocate for less travel-intensive conference practices that can deliver similar benefits. Participants rated the best virtual conferences they had attended to be similarly useful as the average in-person conference, despite the relatively few experiences participants reported, and the relatively recent rise of widespread virtual conferencing. This indicates a great future potential of virtual conferences and underlines the importance of developing and sharing best practices. Participants share our optimism about the future development of virtual communication.

Overall, participants expect their conference experience to worsen somewhat with a switch to virtual (but not hybrid) conferences. However, ECRs belonging to disadvantaged groups expect a switch toward virtual or hybrid conferences to positively affect their groups. Interpretation is complicated by the fact that the same participants did not necessarily expect positive effects for themselves, but it is notable that expectations may differ by group. Future debates on conference formats should explicitly include diverse perspectives, and future research should investigate the differential impacts of different conference formats on specific groups.

While the participants think professional relationships can be maintained virtually, they are divided on whether relationships can be built that way. This finding is in line with the literature (Foramitti et al., 2021; Wenger, 2021), and suggests that preserving the value of conferences in a virtual/hybrid setting depends on careful organization with a focus on facilitating informal interaction and relationship building.

Participants expect to be put at a career disadvantage relative to their peers and seniors only if they alone decide to fly less to conferences, but not if everyone does. This exemplifies the social dilemma nature of climate change and pro-environmental behavior (Milinski et al., 2008). Specifically, it underlines the necessity to make changes collectively in order to change the rules of career success in our community. It also resonates with literature indicating that even highly motivated individuals do not always translate their environmental attitude into pro-environmental behavior due to external constraints (Kollmuss and Agyeman, 2002; Gifford, 2011). Expected career disadvantages may be one such constraint playing an important role.

The survey results indicate that ECRs in environmental psychology view the responsibility for change as a shared responsibility between many different actors. We agree, but wish to emphasize the strategic position of conference organizers as a promising locus of intervention. Conference organizers are situated at the crossroads of relations between stakeholders of the community. They connect internal and external stakeholders, universities, and local agents (e.g., the municipality where the conference is held) and have to harmonize demands coming from all of them. We are aware that organizers operate under constraints, and that organizing conferences comes with a risk, which is exacerbated if a new conference format is implemented without knowing how many community members endorse that format. However, we hope that the data provided here—including qualitative data on participants’ visions for high-quality virtual/hybrid conferences—can offer some orientation and encouragement.

## Limitations

By recruiting participants for our survey through our professional networks, we introduced a sample bias toward participants similar to ourselves. To make our sample more diverse, we directly contacted researchers from regions underrepresented in our networks, but certain countries are still overrepresented. The sample also does not include those who have already left research. Despite our efforts to avoid leading participants’ responses, some social desirability bias may have influenced the data. Also, ECRs may have been more likely to participate if they had strong feelings about conference travel, although we intentionally kept the survey invitation vague. Finally, our sample is too small to draw broader conclusions for ECRs in general, but may permit some generalization for environmental psychology, given the relatively small size of our community.

## Conclusion

Our community case study shows that ECRs in environmental psychology recognize the value of conferences, but have concerns about the impact of current conference travel practices on researchers’ credibility, equity in academia, and the environment. They largely support a shift toward less travel-intensive conference practices including flying less. At the same time, they experience current virtual conferences as less useful than in-person ones, expect a somewhat worse conference experience in the case of a switch toward virtual conferences, and many do not believe they can build professional relationships virtually.

These concerns about both current and alternative conference travel practices must be taken seriously. We call on our community to try hard to face this challenge with optimism. We wish for the community to apply the same creativity we have cultivated in developing psychological behavior change interventions to working to overcome shortcomings of less travel-intensive conference practices.

In particular, this requires a focus on conference travel practices that decrease, not increase, inequalities, and that facilitate informal interaction and networking. To achieve this, we should look to best practices in the wider scientific community (Achakulvisut et al., 2020; Richter et al., 2021; Eidgenössische Technische Hochschule Zürich [ETH], 2022). We further need to build and strengthen the skills needed for virtual community building. Workshops where diverse groups of environmental psychologists and other stakeholders co-develop solutions can ensure that the needs of all stakeholders are considered.

Although devising and implementing less travel-intensive conference practices is a shared responsibility of collective entities and individuals in our field, conference organizers may be particularly well-placed to lead this transformation. We hope this manuscript shows organizers that efforts to reduce conference travel emissions will be welcomed by many ECRs in our field.

## Data Availability Statement

The full survey and dataset presented in this study can be found in an online repository under the following link: https://osf.io/86eay/.

## Ethics Statement

This study involving human participants was reviewed and approved by the ETH Zurich Ethics Commission (EK 2021-N-233). The participants provided informed consent to participate in the study.

## Author Contributions

JK and ASK: conceptualization, methodology, investigation, formal analysis, data interpretation, writing—original draft, writing—review and editing, and visualization. AW: conceptualization, methodology, investigation, formal analysis, data interpretation, writing—review and editing, visualization, and ethics proposal. EK: methodology, investigation, formal analysis, data interpretation, and writing—review and editing. VH, VM, AD, and SL: methodology, investigation, data interpretation, and writing—review and editing. CG, CH, MM, and CR: methodology, investigation, and writing—review and editing. All authors read and approved the final manuscript.

## Conflict of Interest

The authors declare that the research was conducted in the absence of any commercial or financial relationships that could be construed as a potential conflict of interest.

## Publisher’s Note

All claims expressed in this article are solely those of the authors and do not necessarily represent those of their affiliated organizations, or those of the publisher, the editors and the reviewers. Any product that may be evaluated in this article, or claim that may be made by its manufacturer, is not guaranteed or endorsed by the publisher.

## Footnotes

1 We define ECRs as postgraduate researchers, and researchers currently doing their Ph.D. or within 7 years of having completed it, in line with the definition applied by the European Research Council (ERC, 2022).

2
https://osf.io/86eay

3 This includes *n* = 1 person living in the United States and *n* = 1 living in Australia, which self-reported to be part of the Global South, although neither country is part of the Global South according to common definitions (UNDP, 2004).

4 Conference experience was defined as both ease of access to and quality and usefulness of the conference.
