# Supplementary material for: The Need for Sustainability, Equity, and International Exchange: Perspectives of Early Career Environmental Psychologists on the Future of Conferences
Source: Front Psychol. 2022 Jun 16;13:906108. doi: 10.3389/fpsyg.2022.906108 (PMC9245020; doi:10.3389/fpsyg.2022.906108)
Supplement: Supplementary file 1 [file Presentation_1.pdf]

Supplementary Material

1 Supplementary Figures and Tables

1.1 Supplementary Figures

Supplementary Figure S1. How a virtual conference format would affect participants' own conference experience

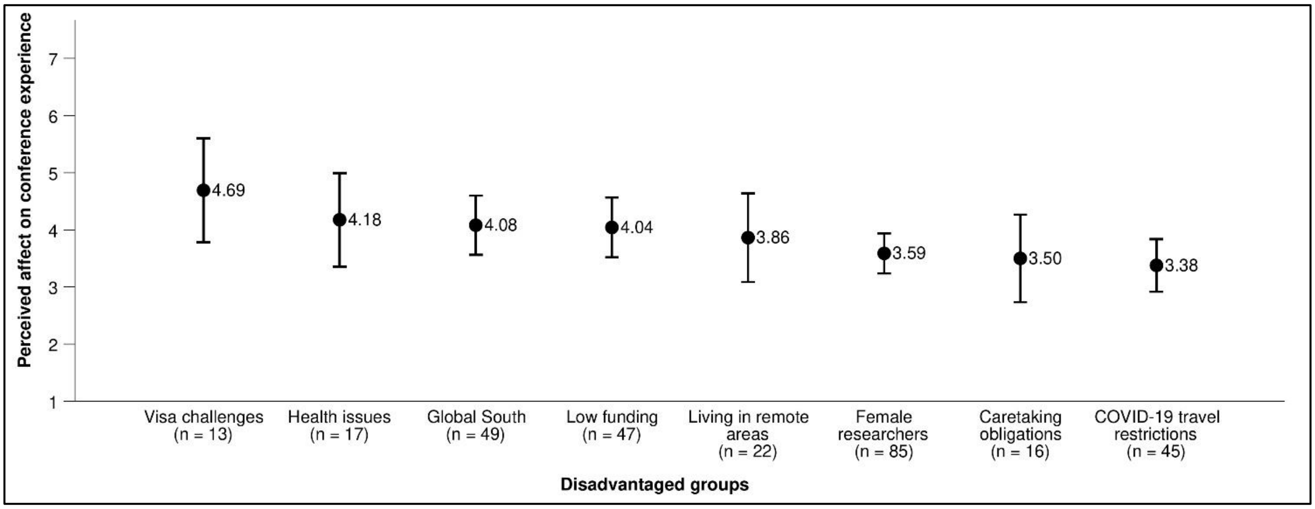

Note. Variables were measured on a scale from 1 *(it would make my experience much worse)* to 7 *(it would make my experience much better).* Error bars represent 95% confidence intervals.

Supplementary Figure S2. How a hybrid conference format would affect participants' own conference experience

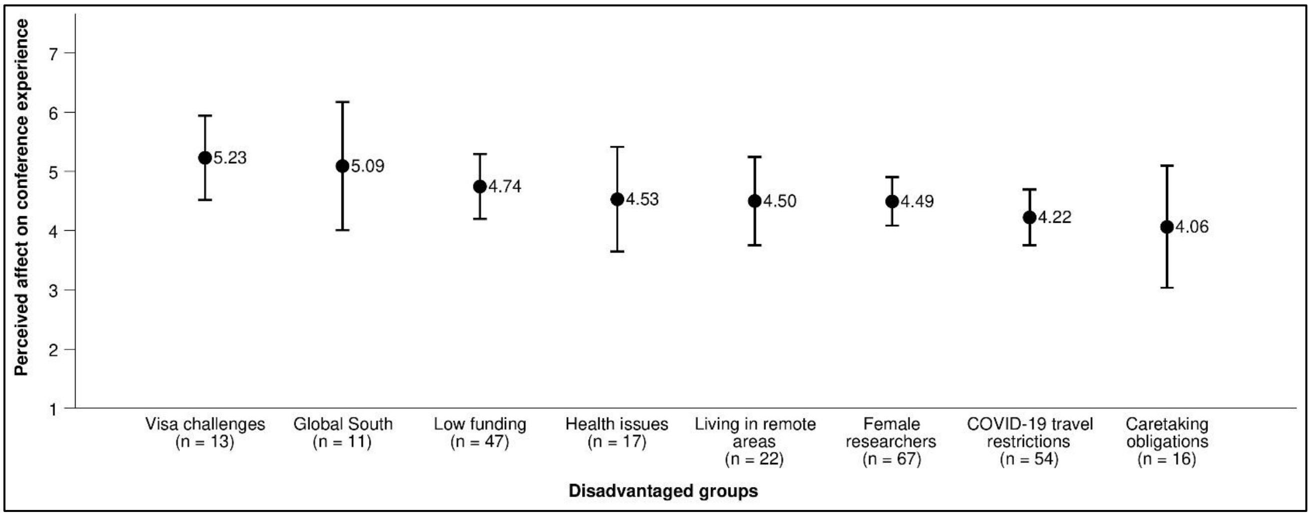

Note. Variables were measured on a scale from 1 *(it would make my experience much worse)* to 7 *(it would make my experience much better).* Error bars represent 95% confidence intervals.

1.2 Supplementary Tables

Supplementary Table S1. Motivations to become a researcher / scientist

Themes	Specifications	Number of cases
Research	- Passion for / enjoyment of / affinity towards research<br>- Way of working<br>- Diversity of (daily) tasks, processes, and methods<br>- Theories, ideas, logic, deep and critical thinking<br>- Flexibility, freedom, and independence<br>- A love for writing / reading / teaching<br>- Intellectual challenge<br>- Enjoying the academic / university environment	52
Changing the world for the better	- Mitigating climate change / Solving environmental problems<br>- Creating real-world impact<br>- Helping to improve the future<br>- Solving societal problems<br>- Generating behavior changes	41
Curiosity / learning	- Curiosity<br>- Wanting to learn / explore	40
Psychology	- Passion for this scientific field<br>- Wanting to understand people / human behavior / society	24
Knowledge production	- Producing / generating knowledge<br>- Shaping / advancing knowledge	12
Other	- Supervisors / mentors<br>- Internationality (e.g., international collaborators)<br>- Prestige / authority<br>- Scientific integrity	5<br>2<br>2<br>1

Supplementary Table S2. Motivations to working on environmental psychology topics

Themes	Specifications	Number of cases
Changing the world for the better	- Mitigating climate change / solving environmental problems / protecting nature<br>- Helping to improve the future<br>- Creating real-world impact<br>- Generating behavior changes<br>- Solving societal problems	77
Psychology	- Wanting to understand human-environment interactions<br>- Wanting to understand people / human behavior / society	32
Other	- Interest in a certain sub-discipline<br>- Supervisors / mentors<br>- A love for / enjoyment of nature<br>- Interdisciplinarity<br>- Randomly	15<br>9<br>9<br>3<br>1

Supplementary Table S3. Participants' visions of sustainable conferences

**Themes**	**Specifications**
Conference format/design	- In-person conferences<br>- Central locations where most of the participants come from (based on the experience of past years)<br>- Smaller conferences with less participants and more specialized<br>- Regional/local conferences<br>- Hybrid conferences<br>- Local/rotating conference hubs across continents / several countries with a virtual connection between them = having multiple conference locations with local meetups<br>- Providing a virtual participation option<br>- Valuing/considering virtual and in-person participants the same<br>- Virtual conferences<br>- Considering time-zone differences<br>- Providing pre-recordings<br>- Recording the Q&A<br>- Adding links with additional information to the presentations<br>- (see Supplementary Table 6 for more ideas on formats and tools used during virtual conferences)<br>- Other designs<br>- Limited participation (a quota on diverse categories of researchers)<br>- Less frequent conferences<br>- Alternating the conference format<br>- Organizing high quality conferences = reducing low effort / low quality conferences
Conference travel/location	- Choosing locations with good train connections<br>- Rewarding or incentivizing train travel<br>- Offsetting travel emissions
Other Measures	- Providing sustainable (e.g., vegan or seasonal) food<br>- Using reusable dishes<br>- Providing/choosing sustainable accommodations<br>- Producing as little waste as possible

Supplementary Table S4. Interactions perceived difficult to conduct virtually

**Themes**	**Specifications**	**Number of cases**
Informal interactions / Socializing	- Chats and informal exchanges<br>- Casual, personal, and private conversations<br>- Social interactions<br>- Getting to know someone / their research interests<br>- Coffee / beverage / lunch / dinner<br>- Spontaneous / unplanned / chance / random encounters<br>- Fun / social activities and events: parties, exploring the area, cultural experiences<br>- Smalltalk / catch-ups<br>- Follow-up discussions	78
Networking with new people	- Networking<br>- Meeting / getting to know new people<br>- Introducing people to each other<br>- Finding collaborators	32
Relationships	- Bonding / making friendships and deeper connections<br>- Building trust/empathy	12
Formal sessions	- Q&As<br>- Poster sessions<br>- Discussing research (results)	8
Other	- Sharing the same space / freely navigating a space<br>- Sitting next to someone / forming groups	6
	- Developing new ideas and being creative	2
	- Reading gestures and body language	2
	- All social interactions	5
	- No social interactions	2

Supplementary Table S5. Interactions perceived easy to conduct virtually

Themes	Specifications	Number of cases
Formal sessions	- Discussing and presenting research (findings)<br>- Poster sessions<br>- Panel discussions<br>- Talks / key notes / symposiums<br>- Q&As and after talk discussions<br>- Formal discussions (on content)<br>- Feedback on presentation	50
Few people	- Small group discussions<br>- One-on-one meetings	15
Informal chats	- Chats<br>- Informal conversations / discussions<br>- Getting to know someone's research interests<br>- Smalltalk / catch-ups	13
Platforms / tools	- Online (team) games<br>- Sending info / links and asking questions via chat boxes<br>- Receiving contact info of other participants (on conference platform)<br>- Twitter	12
Networking with new people	- Finding collaborators<br>- Meeting people in random breakout rooms / speed dating<br>- Introducing people to each other	9
Planned interactions	- Planned / guided discussions (around a specific topic)	8
Other	- No social interactions<br>- All social interactions	7<br>1

Supplementary Table S6. Suggestions and ideas for virtual informal exchange formats

Themes	Specifications
Virtual formats	- Regular networking meetings<br>- Monthly speaker series<br>- Frequent lunchtime talks<br>- Coffee clubs<br>- Answering casual questions<br>- Planned one-on-one / group discussions (on a specific topic)<br>- Scheduled lunch/coffee breaks<br>- Small group meetings<br>- Designated facilitator<br>- Informal communication forums / collaborative spaces to exchange<br>- Sub-threads based on concrete topics<br>- PhD chat channels<br>- Random breakout rooms / speed dating<br>- Virtual writing groups<br>- Interactive virtual workshops<br>- Virtual social activities such as (team) games<br>- Online support meetings to exchange on collaborators and job opportunities, asking for help, answering questions, and providing contact details<br>- E.g. each participant can give a 1min pitch on what they are looking for
Virtual platforms and tools	- Discord<br>- Gather.town<br>- MeetAnyway<br>- Miro boards<br>- Slack<br>- Shared documents and working spaces
Formats and tools for virtual/hybrid conferences	- Planned sessions / ample space for informal exchange/chats<br>- Small group discussions<br>- Short meetings<br>- Using a technology for organic modes of interaction<br>- Planned sessions for networking, finding collaborators, and research ideas<br>- Virtual social activities<br>- Online/party games<br>- Sharing informal photos<br>- Virtual city tours<br>- Conference apps/platforms to contact and chat with other participants<br>- Virtual reality platforms/tools to mingle among participants<br>- Conference/tandem partners<br>- Live surveys for online participants<br>- Recording and sharing answers to open questions from the chat<br>- Virtual, structured poster sessions<br>- Virtual info table

Supplementary Table S7. ‘Other’ responses to the question “Who do you think is responsible for reducing the amount of flying to conferences?”

Categories	Number of mentions
Government / state	8
Policymakers	7
Funding agencies	5
External actors	4
Societal norms	4
Leaders of the scientific field / Supervisors	3
No one	1
Everyone	1

Supplementary Table S8. ‘Other’ responses to the question “Do you count yourself among any of these disadvantaged groups?”

Categories	Number of mentions
LGBTQ+	2
East Asian	1
First generation to college	1
Islander	1
Working class	1

Supplementary Table S9. ‘Other’ responses to the question “Which of the following do you consider compelling reasons why flying to conferences should be reduced in your field?”

Categories	Number of mentions
Pushing land-based travel	4
Financial savings	2
Matching own values	1
Reducing demand on market	1
Reducing stress around this discussion	1
No reason	1
